# The Relationship Between Physical Activity, Emotional Regulation, Psychological Stress, and Mood Among College Students: A Network Analysis Study

**DOI:** 10.3390/bs16050694

**Published:** 2026-05-01

**Authors:** Baole Tao, Zhengwu Li, Jie Han, Tianci Lu, Hanwen Chen, Jun Yan

**Affiliations:** College of Physical Education, Yangzhou University, Yangzhou 225012, China; mz120250810@stu.yzu.edu.cn (Z.L.); mz120250783@stu.yzu.edu.cn (J.H.); dx120240094@stu.yzu.edu.cn (T.L.); dx120230091@stu.yzu.edu.cn (H.C.); yanjun@yzu.edu.cn (J.Y.)

**Keywords:** physical activity, emotion regulation, psychological stress, mood, network analysis

## Abstract

To examine the complex relationships among physical activity, emotion regulation, psychological stress, and mood states in college students, this study analyzed questionnaire data collected from 494 participants. Network analysis was employed to construct a global association network, compare gender differences, and characterize patterns of directed statistical dependencies via directed acyclic graph (DAG) analysis. The results showed that: (1) the network comprised 25 nodes and 94 non-zero edges, reflecting extensive conditional associations across the four domains; (2) bridge centrality analysis identified cognitive reappraisal, self-related emotions, and anger as key bridge nodes, with cognitive reappraisal exhibiting the highest bridge strength; (3) accuracy and stability analyses yielded a centrality stability coefficient (CS) of 0.749 for strength, indicating adequate network stability; (4) network comparison tests revealed no significant gender differences in overall network structure or global strength, although certain local edge weights differed; (5) DAG analysis suggested that stable directional dependencies were primarily concentrated within individual subsystems, with no marked structural differences observed between male and female groups. In conclusion, physical activity, emotion regulation, psychological stress, and mood states appear to constitute an interconnected psychological adaptation system. Cognitive reappraisal, self-related emotions, and anger likely serve as pivotal bridge nodes warranting priority in future longitudinal research and targeted interventions.

## 1. Introduction

The college years constitute a critical developmental period characterized by the transition from adolescence to early adulthood, during which psychological issues are often especially prevalent. College students frequently encounter persistent psychological stress as they navigate academic pressures, social adjustment, career uncertainty, and evolving societal role expectations. Identifying the key psychological and behavioral mechanisms underlying students’ psychological adjustment is therefore essential, with particular emphasis on modifiable factors embedded in daily life. Physical activity is widely recognized as an important behavioral resource for promoting mental health among college students. However, participation is not equally accessible, as social, environmental, economic, and psychological barriers can substantially hinder engagement (Ferreira Silva et al., 2022). Systematic reviews and meta-analyses demonstrate that physical activity or exercise can alleviate symptoms of depression, anxiety, and stress in this population (Huang et al., 2024; J. Li et al., 2025). Nevertheless, significant heterogeneity exists across studies regarding intervention methods, dosages, outcome indicators, and sample characteristics (Stevens et al., 2017). This heterogeneity implies that the effect of physical activity on mental health is unlikely to be simple, homogeneous, or unidirectional; instead, it is more likely embedded within a broader and more complex system of psychological adaptation.

From a mechanistic perspective, emotion regulation may serve as a crucial link between physical activity and mental health. According to the Process Model of Emotion Regulation, cognitive reappraisal is an antecedent-focused strategy that involves reinterpreting a situation to alter its emotional impact, whereas expressive suppression is a response-focused strategy that entails inhibiting the outward expression of an already activated emotion (Cutuli, 2014). Gross and John (2003) proposed that these strategies yield distinct adaptive outcomes: cognitive reappraisal is generally associated with more positive emotional experiences and superior psychological adjustment, while expressive suppression tends to correlate with poorer emotional and social functioning. Recent studies among college students further indicate that better emotion regulation abilities help buffer the accumulation of stress across the semester, while physical activity is linked to improved emotion regulation and better overall mental health (J. Wang et al., 2025). At the same time, individuals experiencing high levels of psychological stress often struggle to mobilize regulatory resources, resulting in compromised negative emotional experiences and behavioral persistence (Demichelis et al., 2024; Hu & Liu, 2025). Consequently, the relationship among physical activity, emotion regulation, and psychological stress is not a simple linear one but may form an interactive network.

In addition to emotion regulation, psychological stress and mood states themselves exhibit multidimensional and dynamic system-level properties (Montpetit et al., 2010). For college students, academic, daily life, developmental, interpersonal, and family pressures frequently coexist; the accompanying mood changes rarely involve isolated fluctuations in a single dimension; rather, they manifest as interrelated shifts across tension, anger, fatigue, depression, diminished vigor, anxiety, and self-evaluation (Ross et al., 1999). Recent research suggests that physical activity is associated not only with reduced stress levels but also with enhanced recovery and improved indices of academic functioning (Teuber et al., 2024). Thus, physical activity may serve as a protective health behavior while remaining susceptible to reciprocal influences from stress perceptions and mood states.

The traditional variable-centered approach, which examines unidirectional relationships between a limited number of constructs solely through regression or mediation models, has limited capacity to reveal the conditional relationships within and between different systems. In recent years, psychological network analysis has offered a novel perspective for understanding complex psychological systems (Briganti et al., 2024). Network theory posits that psychological phenomena are not merely manifest indicators of latent variables but may instead represent dynamic systems formed by the interactions of multiple discrete components (Borsboom, 2017). Within this framework, associations between nodes can facilitate cross-system transmission through local edge connections, while certain bridge nodes may serve as key information relays between distinct psychological systems (Jones et al., 2021). Compared with traditional latent variable models, network analysis is better suited to identify core nodes, bridge nodes, and local connectivity patterns at the specific dimension or item level (Fried & Cramer, 2017); Consequently, it has been increasingly applied to the study of psychopathology, emotionality, and psychological adjustment in college students. Recent studies have employed network methods to examine the relationship between depression and emotion regulation difficulties among college students (Liu et al., 2025), differences in depression, anxiety, and sleep networks under varying stress levels (W. Li et al., 2024), and the integrated relationships between physical activity and variables such as emotion, personality, and motivation (Bi et al., 2026). However, while some studies have focused on psychological symptom networks and others on physical activity networks; few studies have simultaneously incorporated all four categories of variables: physical activity, emotion regulation, psychological stress, and mood states into a single integrative model.

Merely examining bivariate or sequential relationships among physical activity, mental health, emotion regulation, stress, and mood states makes it challenging to elucidate the specific pathways linking physical activity to the psychological adjustment of college students or to identify the precise nodes that serve as critical bridges between systems. Psychological interventions and exercise promotion initiatives for college students typically require precise targeting, especially given limited resources. Identifying key bridge nodes in cross-system transmission may therefore more efficient than implementing generalized interventions that address all variables simultaneously (Jones et al., 2021; Epskamp et al., 2018). Integrating physical activity, emotion regulation, psychological stress, and mood states into a single network framework not only deepens understanding of the organizational structure of the psychological adaptation system in college students but also provides more specific targeting criteria for subsequent integrated psychological and behavioral interventions. Furthermore, gender differences constitute a critical focus in research on psychological adaptation among college students. Previous studies suggest that males and females may differ in levels of physical activity participation (Molanorouzi et al., 2015), emotional expression (Chaplin, 2015), and certain psychological symptom manifestations; However, consistent evidence is currently lacking regarding whether these differences are sufficient to alter the overall network structure linking physical activity, emotion regulation, psychological stress, and mood states. Compared with conventional comparisons of mean differences, network comparison tests allow examination of similarities and differences between groups in terms of overall network structure and global connectivity strength (Epskamp et al., 2018; Bi et al., 2026), thereby offering more detailed structural information for developing gender-sensitive interventions.

Building upon these considerations, the present study employs network analysis to integrate physical activity, emotion regulation, psychological stress, and mood states into a unified analytical framework, thereby systematically examining the overall associative structure of these variables among college students. Specifically, this study aims to: (1) estimate the comprehensive network structure encompassing physical activity, emotion regulation, psychological stress, and mood states; (2) identify key bridge nodes that facilitate cross-system interactions; (3) compare the similarities and differences in network topography and global strength between male and female students; and (4) further explore potential directed statistical dependency patterns among variables using directed acyclic graphs (DAGs). By shifting the focus from aggregate-level relationships to network structure analysis at the dimensional or item level, this study seeks to reveal the intricate organizational patterns of college students’ psychological adaptation system to provide an empirical foundation for integrating mental health interventions.

## 2. Methods

### 2.1. Study Population

This study was conducted in strict accordance with the ethical guidelines of the Declaration of Helsinki and received formal approval from the Ethics Committee of Yangzhou University (No. YXYLL-2022-109) prior to data collection. All participants were briefed on the study’s objectives before the assessment commenced and provided written informed consent. Using convenience sampling, a cross-sectional survey was conducted among undergraduate students at a university in Jiangsu Province, China. A total of 715 initial questionnaires were collected. To ensure the robustness of the network analysis findings, relatively stringent data quality control procedures were implemented. Exclusion criteria comprised: (a) questionnaires with a non-response rate exceeding 30%, a pragmatic data-quality threshold used to exclude cases with substantial missingness that may compromise the stability and interpretability of subsequent analyses (Dong & Peng, 2013); (b) missing key demographic information (e.g., gender or age); and (c) evidence of obvious response patterns or invariant (linearized) answers. After screening, 221 questionnaires were excluded under these criteria, resulting in a final sample of 494 valid responses. Although the exclusion count was relatively high, this primarily reflected the application of stringent data quality screening standards designed to reduce the impact of incomplete or low-quality responses on network estimation. The final sample consisted of 208 males (42.1%) and 286 females (57.9%), with ages ranging from 17 to 21 years (M = 19.27, SD = 1.06).

### 2.2. Research Tools

The Physical Activity Rating Scale-3 (PARS-3) was used to assess the physical activity levels of college students (D. Liang, 1994). This scale consists of three items: physical activity intensity, duration per session, and frequency of activity, each rated on a 1–5 scale. Physical activity volume was calculated using the formula: “intensity × (duration − 1) × frequency”. In this study, the Cronbach’s α coefficient for this scale was 0.803.

The Chinese version of the Emotion Regulation Questionnaire (ERQ), developed by Gross and John (2003) and revised by L. Wang et al. (2007), was used to measure individuals’ trait-level emotion regulation strategies. The scale consists of 10 items, divided into two dimensions: cognitive reappraisal and expressive suppression, with each item rated on a 7 point scale. In this study, the Cronbach’s α coefficient for this scale was 0.783.

The “Chinese College Students’ Psychological Stress Scale” was used to assess individual levels of psychological stress (B. Liang & Hao, 2005). This scale comprises five dimensions: academic, daily life, social, developmental, and family, which reflect stress experiences from different sources. In this study, the scores for each dimension were treated as independent nodes in the network analysis. The Cronbach’s α coefficient for this scale in this study was 0.984.

The Brief Mood State Scale was used to assess individual mood states (Zhu, 1995). The scale comprises seven dimensions: tension, anger, fatigue, depression, vigor, confusion, and self-related emotions. Dimension scores were treated as independent nodes in the network analysis. The Cronbach’s α coefficient for this scale in this study was 0.927.

### 2.3. Data Processing and Statistical Analysis

Data cleaning, descriptive statistics, and reliability analyses were conducted using SPSS (Version 26.0), whereas network analysis and related statistical procedures were performed in R (Version 4.3.1). Indicators of physical activity, emotion regulation, psychological stress, and mood states were included in the network analysis dataset, with gender retained for subgroup comparisons. A total of 25 nodes were ultimately included: 3 indicators for physical activity, 10 items for emotion regulation, 5 dimensions for psychological stress, and 7 dimensions for mood states. Emotion regulation was represented at the item level to preserve the specificity of distinct regulatory strategies, whereas psychological stress and mood states were represented at the dimension level in accordance with their established scale structures. Accordingly, differences in the number or strength of edges across domains may partly reflect differences in measurement granularity rather than substantive differences alone.

The qgraph and bootnet packages in R were employed for network estimation and visualization. The network was estimated using the Extended Bayesian Information Criterion Graphical Least Absolute Shrinkage and Selection Operator (EBICglasso) method. In the resulting visualization, nodes represent variables, while edges reflect partial correlation coefficients between nodes after controlling for all other variables in the network. The thickness of an edge indicates the strength of the association, with solid and dashed lines (or different colors) distinguishing between positive and negative relationships. To facilitate cross-gender comparisons, a consistent layout based on the global network was applied to both the male and female groups.

Bridge centrality indices were estimated using the networktools package to identify nodes that may connect different communities within the network. In accordance with the study’s theoretical framework, the 25 nodes were assigned to four predefined communities: physical activity, emotion regulation, psychological stress, and mood states. Because the estimated network contained negative edges, bridge centrality was evaluated primarily with Bridge Expected Influence (EI), which preserves edge weight signs and is thus more appropriate for signed psychological networks than bridge strength. In the present analyses, Bridge Expected Influence (1-step) served as the primary bridge metric, whereas Bridge Expected Influence (2-step) was treated as supplementary.

Network robustness was assessed with the bootnet package. Edge-weight accuracy was examined through non-parametric bootstrapping (1000 iterations), and centrality stability was evaluated via case-dropping bootstrapping, with the centrality stability (CS) coefficient reported.

The NetworkComparisonTest (NCT) package was employed to examine differences in network topography and global strength between genders. This analysis tested for network structure invariance and global strength. Edge-level differences were reported to avoid inflated Type I error from multiple comparisons.

Directed Acyclic Graphs (DAGs) were estimated using the hill-climbing algorithm in the bnlearn package (5.1), with the Bayesian Information Criterion (BIC) as the scoring function, to explore directed statistical dependencies. Bootstrap resampling (with edge stability thresholds) was applied to retain only stable edges in the final DAGs. Given the cross-sectional design, directed edges were interpreted as patterns of statistical dependency rather than causal or temporal relationships. Separate DAGs were generated for male and female groups, and Structural Hamming Distance (SHD) was calculated to quantify the structural differences between them.

## 3. Results

### 3.1. Descriptive Statistics

The final network analysis encompassed 25 nodes: 3 indicators from the Physical Activity Rating Scale-3, 10 items from the Emotion Regulation Questionnaire, 5 dimensions from the Chinese College Students’ Psychological Stress Scale, and 7 dimensions from the Brief Mood State Scale. Descriptive statistics, including means and standard deviations for all variables, are presented in Table 1.

### 3.2. Overall Network

#### 3.2.1. Overall Network Estimation

**Figure 1:**
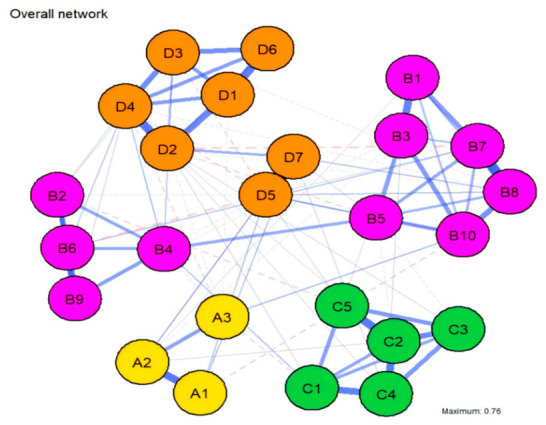
Overall Network Structure. Nodes represent individual variables, and edges represent partial correlation coefficients between nodes after controlling for all other variables in the network. Edge thickness reflects the magnitude of the association, with thicker edges indicating stronger relationships. Solid lines denote positive correlations, while dashed lines denote negative correlations. Node groupings: A1–A3 = physical activity; B1–B10 = emotion regulation; C1–C5 = psychological stress; D1–D7 = mood states.

A Gaussian Graphical Model (GGM) comprising 25 nodes was estimated using the EBICglasso regularization algorithm (Figure 1). The distribution of edge weights within the network revealed that robust associations were primarily concentrated within each community (intra-community edges), whereas inter-community connections were relatively sparse. The strongest edge in the network was identified between vigor (D5) and Self-related Emotions (D7) (r = 0.799), followed by the association between two cognitive reappraisal items, B8 (“I regulate my emotions by changing my perspective on the situation”) and B7 (r = 0.547). Other prominent associations included Anger (D2) and Depression (D4) (r = 0.464), Life Stress (C2) and Family Stress (C5) (r = 0.439), and Exercise Intensity (A1) and Duration (A2) (r = 0.432). Furthermore, substantial connection strengths were observed between Tension (D1) and Confusion (D6) (r = 0.430), Life Stress (C2) and Developmental Stress (C4) (r = 0.420), Academic Stress (C1) and Developmental Stress (C4) (r = 0.381), cognitive reappraisal items B1 and B3 (r = 0.370), and Life Stress (C2) and Social Stress (C3) (r = 0.356). In addition to these predominantly positive associations, a small number of negative edges were also observed. In particular, cognitive reappraisal item (B7) showed a weak negative association with anger (D2) (r = −0.078), and depression (D4) showed a weak negative association with vigor (D5) (r = −0.068). Although these negative edges were relatively weak, they are theoretically consistent with the possibility that greater use of adaptive reappraisal is associated with lower anger, and that depressive mood is inversely related to vigor.

Overall, high-weight edges within the network were predominantly concentrated within their respective variable systems. Specifically, within the mood state community, robust associations were identified between vigor (D5) and Self-related Emotions (D7), Anger (D2) and Depression (D4), and Tension (D1) and Confusion (D6). Similarly, within the emotion regulation system, connections between cognitive reappraisal items (B7–B8, B1–B3, B6–B9, and B8–B10) were particularly prominent. In the psychological stress community, Life Stress (C2) exhibited strong links with Family Stress (C5), Developmental Stress (C4), and Social Stress (C3). Regarding physical activity, the association between Intensity (A1) and Duration (A2) was the most pronounced. These patterns suggest that the core topology of the network is characterized by high internal coupling within each domain. Correspondingly, cross-system connections did not dominate the strongest edges. This indicates that interactions among the four variable domains rely more on the local bridging effects of specific key nodes rather than on pervasive, uniform cross-domain connectivity.

#### 3.2.2. Bridge Centrality and Sensitivity Analyses

Because the estimated network contained negative edges, bridge centrality was evaluated primarily using Bridge Expected Influence (EI), which preserves the sign of edge weights and is therefore more appropriate than bridge strength for signed networks. Bridge Expected Influence (1-step) was treated as the primary bridge index, whereas Bridge Expected Influence (2-step) was used as a supplementary indicator. The results showed that cross-community bridging was limited and concentrated in only a small number of nodes. As presented in Table 2, B8 and self-related emotions (D7) exhibited the largest positive one-step bridge expected influence values (both = 0.081), whereas B7 and anger (D2) exhibited the largest negative one-step bridge expected influence values (both = −0.078). These findings indicate that cross-domain bridging in the present network was not broadly distributed across nodes, but instead concentrated in a very limited set of variables. As shown in Table 2, the reduced/sensitivity network yielded a highly similar bridge pattern to that of the original network, with self-related emotions and B8 retaining the largest positive one-step bridge expected influence values, and vigor remaining non-prominent in the primary bridge analysis. In particular, self-related emotions and specific cognitive reappraisal items appeared to play a more prominent role in connecting communities than did most mood-state or physical-activity indicators. Although vigor was strongly embedded within the mood-state subsystem, it did not emerge as a prominent bridge node in the primary one-step bridge analysis.

#### 3.2.3. Network Accuracy and Stability

**Figure 2:**
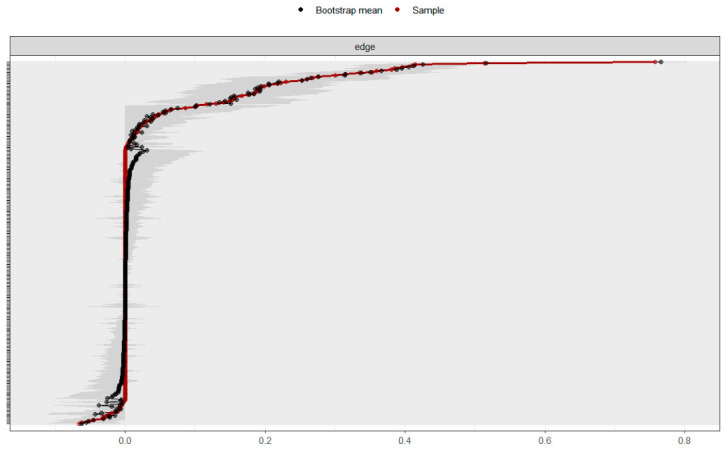
Bootstrap results for overall network edge weights. The figure displays the bootstrap estimates of the network edge weights. Black lines represent the sample estimates, and the gray areas denote the 95% bootstrap confidence intervals. Narrower confidence intervals indicate higher precision in the edge weight estimates.

**Figure 3:**
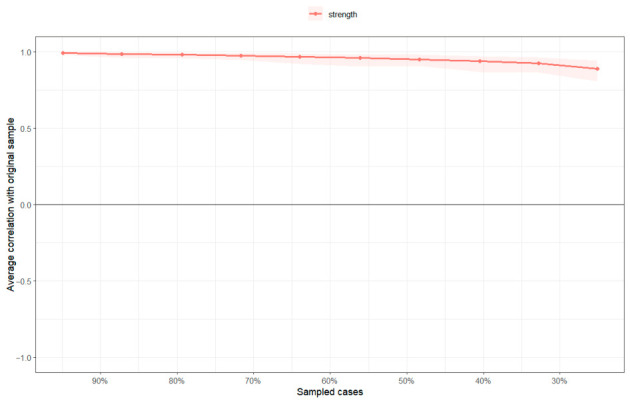
Estimated Stability of Overall Network Centrality. This figure depicts the correlation between the original centrality indices and those derived from sub-sampled datasets. The horizontal axis represents the proportion of the sample dropped, and the vertical axis represents the correlation coefficient. A curve that remains near 1.0 as the dropout rate increases indicates superior stability of the corresponding centrality metric.

The accuracy of the network parameters was evaluated using 1000 nonparametric bootstrap resamples. In the present network, edge weights represent regularized partial correlation coefficients, with values closer to zero indicating weaker conditional associations and larger absolute values indicating stronger associations after controlling for all other nodes. Because no universally accepted cutoff values exist for classifying edge weights as weak, moderate, or strong in psychological network analysis, these estimates were interpreted comparatively within the network and in conjunction with their bootstrap confidence intervals. As shown in Figure 2, edge weight estimates, especially those with larger magnitudes, remained relatively stable across resamples, while lower-weight edges exhibited wider confidence intervals, indicating greater sampling uncertainty. Regarding network stability, a case-dropping bootstrap procedure was employed to assess the robustness of node strength. The results indicated that the Centrality Stability (CS) coefficient for node strength was 0.749, well exceeding the recommended threshold of 0.50 (Epskamp et al., 2018), which confirms that the centrality rankings remained stable even after dropping a substantial proportion of the sample (Figure 3).

### 3.3. Gender Differences in Networks

#### 3.3.1. Network Estimation

**Figure 4:**
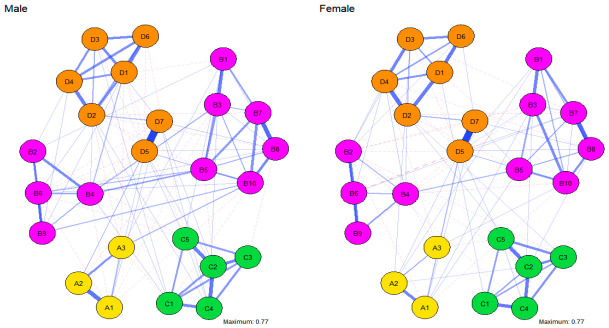
Network diagrams for the male and female groups. Nodes represent individual variables, and edges denote partial correlation coefficients between nodes after controlling for all other variables in the network. Edge thickness reflects the magnitude of the association. Solid lines denote positive correlations, while dashed lines denote negative correlations.

To examine gender-specific variations in network architecture, 25-node Gaussian Graphical Models (GGMs) were estimated separately for male and female groups (Figure 4). The Network Comparison Test (NCT) with 1000 permutations was then employed to assess potential differences in network structural invariance and global strength. The NCT results revealed no significant difference in the overall network structure between the male and female groups (M = 0.22, *p* = 0.436). Similarly, the comparison of global connectivity strength did not reach statistical significance (GS male = 11.56, GS female = 10.70, S = 0.86, *p* = 0.137). These findings suggest that the interrelationships among physical activity, emotion regulation, psychological stress, and mood states are consistent across genders in the current sample.

#### 3.3.2. Comparison of Gender Networks

**Figure 5:**
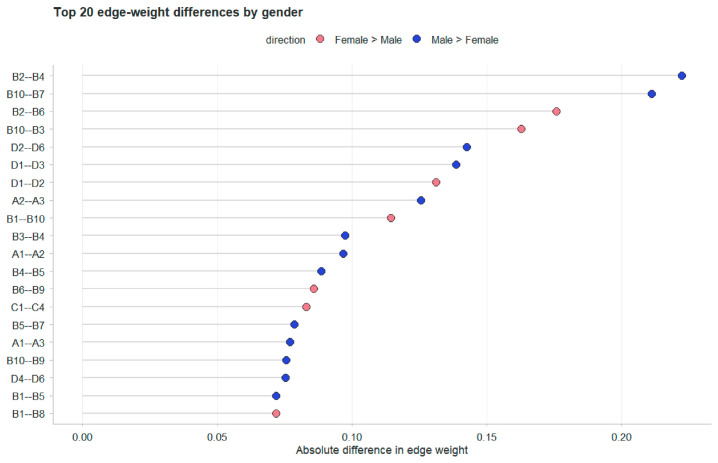
Comparison of the top 20 edges with the largest weight differences between genders. The horizontal axis represents the absolute difference in edge weights. Red bars indicate stronger connectivity in the female group, while blue bars indicate stronger connectivity in the male group.

**Figure 6:**
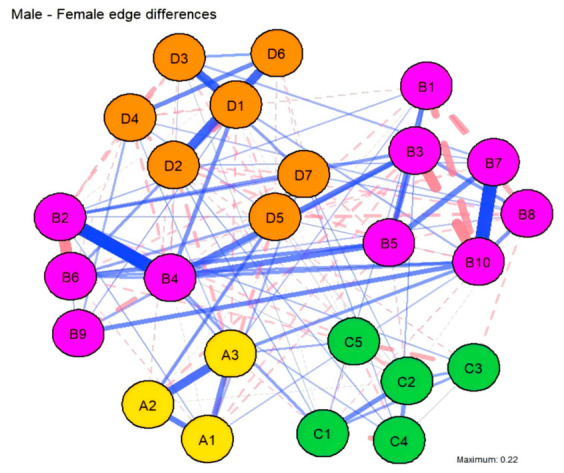
Differences in Network Edge Weights Between Male and Female College Students. The solid blue lines indicate stronger influence/connectivity in the male group, whereas dashed pink lines indicate stronger influence/connectivity in the female group. Edge thickness is proportional to the magnitude of the difference between the groups.

To explore gender-related nuances in local connectivity, the 20 edges exhibiting the largest absolute differences in weights between the male and female groups were identified and ranked (Figure 5 and Figure 6). Within the female group, the most prominent edges included Expressive Suppression (B2–B6, 0.33 vs. 0.00; B6–B9, 0.37 vs. 0.27), Cognitive Reappraisal (B10–B3, 0.29 vs. 0.00), Academic–Developmental Stress (C1–C4, 0.44 vs. 0.29), and Tension–Anger (D1–D2, 0.37 vs. 0.25). In contrast, the male group displayed stronger associations within Expressive Suppression (B2–B4, 0.31 vs. 0.00; B4–B5, 0.26 vs. 0.00), Cognitive Reappraisal (B10–B7, 0.24 vs. 0.00), and the Tension–Fatigue (D1–D3, 0.28 vs. 0.13) connection.

Distributionally, gender variations were predominantly concentrated within the emotion regulation system, specific mood states, and a limited number of cross-domain interactions. Specifically, females exhibited more robust associations in the Anger–Self-related Emotions (D2–D7) and Family Stress–Self-related Emotions (C5–D7) pathways, while males showed stronger links between Expressive Suppression (B4) –Tension (D1) and Anger–Confusion (D2–D6). It is important to note that these localized discrepancies are primarily descriptive. Given that the NCT showed no significant differences in global structure or connectivity, these patterns should be viewed as variations in specific path intensities rather than a fundamental gender-based differentiation in the underlying psychological mechanisms.

### 3.4. Directed Acyclic Graphs

#### 3.4.1. General Directed Acyclic Graphs

**Figure 7:**
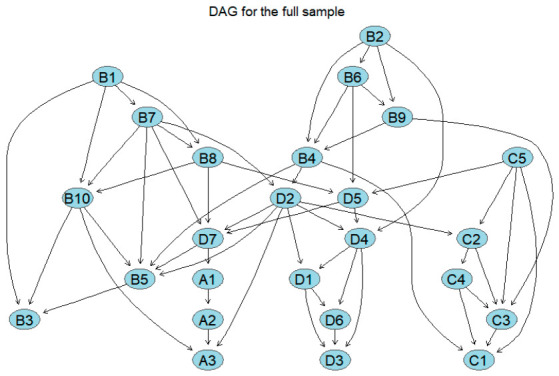
Original directed acyclic graph of the entire sample. The arrows in the figure indicate directional dependencies between variables.

To examine the directional dependency patterns among the 25 nodes, this study employed the full dataset to derive an initial directed acyclic graph (DAG; Figure 7) via a hill-climbing algorithm based on a Gaussian BIC score criterion. The resulting structure comprises 25 nodes and 55 directed edges, suggesting a degree of complexity in the directional associations among the variables. Within the physical activity subsystem, a linear pathway emerged, directed from intensity (A1) to single-session duration (A2) and subsequently to frequency (A3); similarly, the emotion regulation, psychological stress, and mood state subsystems exhibited relatively dense directed connections. However, the initial DAG estimated from a single sample is susceptible to sample-specific idiosyncrasies; consequently, these results should be regarded as exploratory and cannot be directly interpreted as robust directional conclusions. To mitigate the influence of sample-specificity on the structural learning outcomes, this study further integrated bootstrap resampling results to identify relatively robust pathways based on the frequency of occurrence and directional stability of the directed edges.

**Figure 8:**
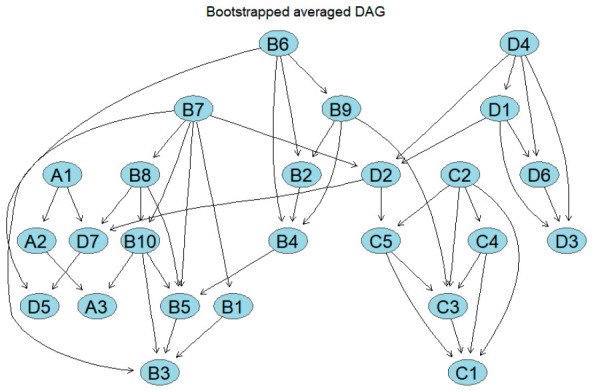
Directed acyclic graph of the overall network, averaged via bootstrapping. The figure shows only those paths that appeared frequently and exhibited relatively stable directions across repeated sampling.

To examine the directional dependency patterns among the 25 nodes, this study developed a relatively stable directed acyclic graph (DAG; Figure 8) derived from 1000 bootstrap resamples. Compared to the initial model, the number of retained directed paths decreased significantly, suggesting that under stricter filtering conditions, stable directional relationships are primarily concentrated within a small number of core dependency chains. Within the physical activity subsystem, the linear pathways of intensity (A1) → single-session duration (A2) (strength = 1.000, direction = 0.525) and single-session duration (A2) → monthly exercise frequency (A3) (strength = 0.994, direction = 0.856) were stably retained. Within the emotion regulation subsystem, multiple directed connections were retained, specifically B7 → B1, B7 → B8, B8 → B10, B10 → B3, B10 → B5, B6 → B2, B6 → B9, B9 → B4, and B4 → B5, indicating a relatively clear local directed structure. In the psychological stress subsystem, life stress (C2) consistently predicted social stress (C3), developmental stress (C4), and family stress (C5); subsequently, developmental stress (C4) and family stress (C5) were directed toward academic stress (C1) and social stress (C3), suggesting that the stress subsystem exhibits a directed association pattern wherein life, developmental, and family stress function as antecedent nodes. In the mood state subsystem, depression (D4) was consistently directed toward tension (D1), anger (D2), fatigue (D3), and panic (D6), with tension (D1) further predicting anger (D2), fatigue (D3), and panic (D6). Additionally, stable associations were observed between anger (D2) and self-related emotions (D7), as well as between panic (D6) and fatigue (D3). These results indicate that stably retained directed pathways are primarily concentrated within their respective subsystems.

#### 3.4.2. Comparison of Gender-Specific DAGs

**Figure 9:**
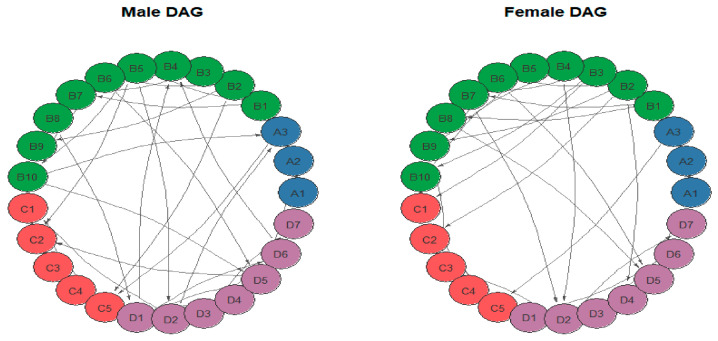
Directed acyclic graphs for the male and female groups. The **left** figure shows the DAG for the male group, and the **right** figure shows the DAG for the female group.

Building upon the relatively stable directed acyclic graphs (DAGs), this study estimated group-specific DAGs for male and female cohorts (Figure 9) to examine potential gender differences in directional dependency structures. Visual inspection indicated that the networks across both groups exhibited a high degree of similarity in subsystem clustering and pathway patterns; specifically, both cohorts retained several local linear structures within the physical activity, emotion regulation, psychological stress, and mood state subsystems. To formally quantify the differences in directional structures between the two groups, we calculated the Structural Hamming Distance (SHD) between the respective networks. Results indicated a Structural Hamming Distance of 61 between the DAGs of the male and female groups. According to a null distribution generated via 1000 permutation tests, this observed value did not reach statistical significance (*p* = 0.184). These findings suggest that, within the current sample, there are no significant differences between male and female undergraduates in the global structure of their directional dependency networks. This finding aligns with the previous comparisons of undirected networks, indicating that the two groups share a high degree of similarity in the overall organizational patterns underlying the networks comprising these four categories of variables.

## 4. Discussion

This study examined the relationships among physical activity, emotion regulation, psychological stress, and mood states in college students from a network analysis perspective. Results indicated that these four domains do not constitute a collection of independent variables, but rather form an interconnected system with a distinct structural organization. Within the global network, stronger connections were primarily concentrated within their respective subsystems, whereas cross-subsystem links relied more heavily on a limited number of specific bridge nodes. These findings suggest that psychological adaptation in college students emerges not from any single variable but from the combined effects of several functional subsystems that are highly integrated internally yet interconnected through localized pathways (Fan et al., 2024; Lazarus, 1984). In other words, physical activity, emotion regulation, psychological stress, and mood states each possess a cohesive internal structure while simultaneously enabling cross-subsystem transmission via targeted bridging mechanisms. This interpretation aligns with network theory, which views psychological phenomena “consist of systems composed of interacting components” (Borsboom et al., 2021). Previous research has demonstrated associations between physical activity and reduced depression, anxiety, and stress among college students; however, such links are unlikely to reflect simple unidirectional main effects and may instead depend on broader constellations of regulatory, contextual, and affective processes (Huang et al., 2024; Teuber et al., 2024). Accordingly, situating physical activity within a larger adaptive system offers a more realistic account of psychological functioning than conceptualizing it solely as an external protective factor.

The present findings further revealed that cross-subsystem bridging was limited and concentrated in only a small number of nodes rather than being broadly distributed across the network. Specifically, B8 (“I regulate my emotions by changing my perspective on the situation”) and self-related emotions exhibited the largest positive one-step bridge expected influence values, whereas B7 (“When I want to feel more positive emotions, I change the way I view the situation”) and anger showed the largest negative values. This pattern suggests that inter-systemic information transmission is not evenly distributed but instead depends on a highly restricted set of bridge nodes. Theoretically, this accords with the concept of bridge nodes in psychological network analysis, which posits that only certain nodes serve as key conduits linking relatively distinct communities and shaping cross-domain influence (Jones et al., 2021).

Among these nodes, self-related emotions emerged as one of the most prominent positive bridges, indicating that self-evaluative and self-referential emotional experiences may help connect mood states with other psychological subsystems, consistent with previous work highlighting the integrative role of self-conscious and self-referential emotional processing in broader psychological functioning (Tracy et al., 2007; Herbert et al., 2011). The finding implies that self-related affect functions not only as an internal emotional outcome but also as a pathway linking regulatory and behavioral processes. Similarly, B8 displayed the strongest positive bridge expected influence, suggesting that this particular cognitive reappraisal tendency may serve as an important connector across communities. Given that both B8 and B7 derive from the cognitive reappraisal dimension of the Emotion Regulation Questionnaire, the pattern aligns with the established view that reappraisal constitutes a core antecedent-focused strategy with significant implications for affective functioning (Gross, 1998; Gross & John, 2003). Collectively, these results indicate that cognitive reappraisal is not uniformly relevant across systems; rather, specific components appear more central than others in linking emotional states with adjacent domains. By contrast, B7 and anger exhibited the largest negative one-step bridge expected influence values, demonstrating that not all bridge effects operate in a positive or mutually reinforcing direction. In particular, anger as a high-arousal negative mood state, may represent an antagonistic or inhibitory pathway in cross-community dynamics. Likewise, B7 appeared to play a negative bridging role, suggesting that this more goal-directed, positivity-oriented reappraisal component may be inversely associated with activation in other communities. Taken together, these observations imply that bridge nodes in the present network should not be viewed simply as facilitators of global connectivity; instead, some may reflect suppressive, counterbalancing, or inverse cross-domain associations, consistent with the broader bridge-node perspective in psychological network analysis (Jones et al., 2021).

Network comparison analysis revealed no significant differences between male and female students in global network structure or overall connectivity strength. This suggests that, in the present sample, the overall relationships among physical activity, emotion regulation, psychological stress, and mood states are highly consistent across genders. Nevertheless, fine-grained comparisons of local edge weights identified differences in several discrete connections. These findings suggest that gender-based variations are more likely to manifest as shifts in the magnitude of specific emotion regulation pathways or isolated cross-domain edges, rather than as a wholesale reorganization of the global network architecture. This interpretation aligns with the emerging consensus in the psychological network literature, where group differences typically appear in local edge weights or bridging pathways rather than through fundamental structural divergence (Bi et al., 2026).

Analysis of directed acyclic graphs (DAGs) offers additional insight into potential directional dependencies among variables. The bootstrap-averaged DAG indicated that relatively stable directional relationships predominate within individual subsystems, whereas inter-systemic directional edges show limited stability. This pattern parallels the undirected network results, suggesting that interactions across the four domains more likely represent conditional associations than robust cross-domain directional sequences in cross-sectional data. Consequently, the DAG findings should be interpreted as exploratory indicators of statistical dependencies rather than conclusive evidence of causal pathways (Rohrer, 2018). In cross-sectional designs in particularly, edges appearing in ostensibly upstream or downstream positions within the DAG may still be confounded by unobserved variables, ambiguous temporal ordering, or sampling variability. Nonetheless, the DAG structure suggests that while local linear structures exist within subsystems, cross-system influences rarely manifest as stable, singular pathways, but instead emerge through diffuse transmission across multiple nodes (Epskamp et al., 2018). This underscores the need for longitudinal designs and experimental interventions to clarify the genuine dynamic mechanisms underlying psychological adaptation in college students.

From a practical standpoint, the present findings suggest that interventions aimed at promoting psychological adaptation among college students may be more effective when adopting an integrated systems perspective. Rather than targeting physical activity, stress, and mood in isolation, efforts may benefit from prioritizing key bridge nodes that connect these domains. In the current network, cognitive reappraisal strategies, self-related emotions, and anger management emerged as potentially important intervention targets. Teaching students to reframe stressful situations—thereby modulating self-perception and mitigating hostile affect—may thus yield broader benefits across the system. Although no causal inferences can be drawn from these cross-sectional data, the results may help inform the development of more focused and coordinated health promotion programs that integrate cognitive-behavioral techniques for college students.

## 5. Limitations

This study has several limitations that warrant careful consideration when interpreting the estimated edges and bridge indices. First, the cross-sectional design means that the network reflects patterns of between-person conditional associations rather than causal relationships or within-person processes. Second, although Bridge Expected Influence (EI) was used as the primary metric to accommodate negative edges, the bridge results remain conditional on the specific node set, the chosen bridge metric for signed networks, and the a priori community structure. In the present data, cross-community bridging was limited and concentrated in only a small number of nodes. Third, the network did not incorporate several potentially relevant health-related, behavioral, and contextual variables, such as sleep quantity and quality, pain or nonspecific fatigue, medication use, sport participation or exercise identity, self-efficacy, and academic or socioeconomic context. Omission of these variables may have influenced the estimated edges and bridge indices in this cross-sectional psychometric network. Fourth, several mood-related nodes (e.g., vigor, fatigue, depression, tension/stress, and self-related emotions) may partly overlap in affective or arousal content, and measurement error could further affect the observed network structure. Finally, the four domains were represented at different levels of aggregation, with emotion regulation modeled at the item level and psychological stress and mood states at the dimension level; such differences in measurement granularity may have influenced edge density and bridge estimates. Future research should include broader covariate assessment, formally evaluate potential node redundancy, and examine the robustness of these findings through longitudinal or intensive repeated-measures designs.

## 6. Conclusions

The present study utilized a network analytic approach to systematically elucidate the complex interplay among physical activity, emotion regulation, psychological stress, and mood states among college students. The results revealed a dense architecture of conditional associations across the four domains, with pronounced local clustering within each subsystem. Bridge centrality analysis demonstrated that inter-systemic connectivity is not uniformly distributed but is channeled through specific functional hubs, notably cognitive reappraisal, self-related emotions, and anger. Robustness checks confirmed that the global network architecture exhibited high levels of accuracy and stability. Comparative analyses further demonstrated invariance in global network structure and strength across genders, along with broad consistency in directed acyclic graph (DAG) topologies, although some differences emerged in local edge weights. In conclusion, physical activity, emotion regulation, psychological stress, and mood states form a cohesive and interconnected psychological adaptation system in college students. Cognitive reappraisal, self-related emotions, and anger function as pivotal bridge nodes. These findings highlight the value of prioritizing such hubs in future longitudinal research and in the development of targeted, systems-oriented interventions.

## Figures and Tables

**Table 1:** Descriptive statistics for each node.

Dimension	Node	Variable	M	SD
physical activity	A1	Exercise intensity	1.94	1.026
	A2	Duration of a single exercise session	2.96	1.156
	A3	Frequency of exercise per month	3.08	0.995
Emotion Regulation (Cognitive Reappraisal)	B1	When I want to feel more positive emotions, I change my way of thinking	5.19	1.279
	B3	When I want to reduce negative emotions, I change my way of thinking	4.09	1.426
	B5	When facing stressful situations, I think in ways that help me stay calm	4.99	1.312
	B7	When I want to feel more positive emotions, I change the way I view the situation	3.72	1.448
	B8	I regulate my emotions by changing my perspective on the situation	4.86	1.269
	B10	When I want to reduce negative emotions, I change the way I view the situation	3.88	1.474
Emotion Regulation (Expressive Suppression)	B2	I keep my emotions from showing	4.95	1.213
	B4	When I feel positive emotions, I am careful not to show them	4.87	1.209
	B6	I control my emotions by not expressing them	3.73	1.344
	B9	When I feel negative emotions, I make sure not to show them	4.76	1.192
Psychological stress	C1	Learning Dimension	67.2	22.225
	C2	Life Dimension	64.48	29.211
	C3	Social Dimension	35.08	17.395
	C4	Development Dimension	102.28	36.838
	C5	Family Dimension	39.89	26.451
Mood State	D1	Stress Dimension	12.74	4.937
	D2	Anger Dimension	13.52	5.858
	D3	Fatigue Dimension	10.71	4.281
	D4	Depression Dimension	11.68	5.248
	D5	Vigor Dimension	18.45	4.684
	D6	Panic Dimension	10.8	4.011
	D7	Self-Related Emotional Dimensions	14.06	3.385

**Table 2:** Sensitivity analysis of bridge expected influence: original vs reduced network.

Node	Community	Original Bridge EI (1-Step)	Reduced Bridge EI (1-Step)	Original Bridge EI (2-Step)	Reduced Bridge EI (2-Step)	Interpretation
B8	Emotion regulation	0.081	0.081	0.115	0.116	Positive bridge influence remained stable
D7	Mood states	0.081	0.081	0.136	0.136	Positive bridge influence remained stable
B7	Emotion regulation	−0.078	−0.089	−0.107	−0.111	Negative bridge influence remained stable
D2	Mood states	−0.078	−0.089	−0.135	−0.136	Negative bridge influence remained stable

## Data Availability

Please contact the corresponding authors to obtain the data.
